# Aberrant systemic acute-phase complement responses in conjunction with soluble CR1 attribute to varying grades of dengue disease severity

**DOI:** 10.3389/fimmu.2025.1731011

**Published:** 2026-01-16

**Authors:** Abdul R. Anshad, Shanmugam Saravanan, Amudhan Murugesan, Ravindran Vighnesh, Sivadoss Raju, Rajendran Kannan, Yean K. Yong, Marie Larsson, Esaki M. Shankar

**Affiliations:** 1 Infection and Inflammation, Department of Biotechnology, Central University of Tamil Nadu, Thiruvarur, India; 2 Saveetha Institute of Medical and Technical Sciences, Chennai, India; 3 Department of Microbiology, Government Theni Medical College and Hospital, Theni, India; 4 Department of Biotechnology, Anna University, Chennai, India; 5 State Public Health Laboratory, Directorate of Public Health and Preventive Medicine, Chennai, India; 6 Laboratory Center, Xiamen University Malaysia, Sepang, Malaysia; 7 Molecular Medicine and Virology, Department of Biomedical and Clinical Sciences, Linköping University, Linköping, Sweden

**Keywords:** acute-phase responses, complement, dengue, platelets, sCR1, uric acid

## Abstract

**Background:**

Dengue virus (DENV) infection poses a serious health burden across the tropical and sub-tropical regions. The role of complement factors and acute-phase reactants in clinical dengue remains ambiguous.

**Methods:**

The cross-sectional study enrolled 156 participants, with 114 confirmed clinical dengue cases and 42 healthy controls. Serological profiling (NS1, anti-DENV IgM, and anti-DENV IgG), estimation of serum acute-phase reactants, clinico-laboratory parameters, and viral load were performed to classify dengue patients under dengue with warning signs (DWS+, *n* = 35), dengue without warning signs (DWS-, *n* = 74), and severe dengue (*n* = 5) (based on varying grades of severity) in accordance with the 2009 WHO guidelines. Measurement of complement factors, i.e., C1 inhibitor (C1Inh) (*n* = 145), C1q (*n* = 152), C2 (*n* = 146), C3a (*n* = 153), C3b (*n* = 152), mannose-binding lectin (MBL) (*n* = 151), C5a (*n* = 150), and soluble complement receptor 1 (sCR1, also designated as sCD35) (*n* = 139), was performed using commercial ELISA, and their concentrations were correlated with acute-phase reactants, clinical laboratory parameters, grades of dengue severity, and platelet levels.

**Results:**

Our analysis showed a significant alteration in early classical complement proteins, C1Inh, C1q, and C2. The levels of downstream factors and sCR1 remained largely unchanged across both the grades of dengue severity and primary/secondary dengue cohorts. Univariate analysis revealed NS1 positivity, IgG positivity, age, urea, and sCR1 as factors associated with disease severity. Our multivariate analysis showed sCR1 as the only independent predictor that correlated negatively with dengue severity. Every unit increase of sCR1 was associated with 22% reduced odds of dengue severity. Platelet counts showed a negative association with red cell distribution width (RDW) and basophils and a strong positive correlation with serum uric acid levels.

**Conclusions:**

Our findings show that aberrant complement activation and levels of sCR1 could attribute to varying grades of dengue severity. Given its inverse association, the levels of sCR1 could likely render early prediction of dengue disease severity. The role of sCR1 in complement-mediated pathogenesis in dengue remains a gray area of investigation.

## Introduction

Dengue virus (DENV), transmitted by female *Aedes* mosquitoes, represents a challenging global public health concern (1, 2). Globally, an estimated 3.9 billion people have become infected, mainly in the tropical and sub-tropical regions, with an eightfold increase in incidence reported in the last two decades (3). Notwithstanding that DENV infections are often asymptomatic, exacerbation could result in severe dengue (4). Four antigenically distinct serotypes, i.e., DENV1–DENV4, circulate in the population, while a fifth sylvatic serotype reportedly circulate among non-human primates (5, 6).

The disease spectrum and severity are protean and may vary from unapparent infection to life-threatening severe dengue, often marked by severe thrombocytopenia and multi-organ failure (7). Progression of the disease from mild to severe dengue is influenced by various factors like heterologous virus serotype and host inflammatory responses (8, 9). The 1997 WHO classification stratified dengue into dengue fever (DF), dengue hemorrhagic fever (DHF), and dengue shock syndrome (DSS), with a DF diagnosis emphasizing the requirement of laboratory confirmation. Furthermore, as per the revised 2009 WHO classification, dengue infection is also categorized (based on disease severity) as dengue with warning signs (DWS+), dengue without warning signs (DWS-), and severe dengue. DWS+ is characterized by symptoms/signs, including abdominal pain, recurrent vomiting, lethargy, hepatomegaly, hemoconcentration, and thrombocytopenia, whereas severe dengue includes severe plasma leakage, hemorrhage, and organ failure (10).

Of the various inflammatory pathways, the complement activation cascade holds key in dengue pathogenesis, and its role in clinical dengue largely remains obscure (11). The complement system bridges the innate and the adaptive immune systems (12, 13) via three major pathways, namely, the classical, the alternative, and the lectin pathways (14), and also the blood coagulation pathway. Complement activation culminates in heightened plasma levels of anaphylatoxins (especially C3a, C4a, and C5a), driving the infiltration of polymorphonuclear neutrophils (PMNs) and natural killer (NK) cells, leading to excessive release of pro-inflammatory cytokines, which, at times, could entail endothelial dysfunction (i.e., vasodilation and increased vascular permeability) (15).

The complement system appears to play a role in dengue pathogenesis by inducing inflammation and tissue/organ damage. The mannose-binding lectin (MBL) pathway appears to limit viral replication in asymptomatic (self-limiting) and primary dengue (16). Elevated complement anaphylatoxins have been reported in severe secondary dengue, whereas soluble immune complexes during acute dengue disease will become bound by CR1 (CD35) expressed on erythrocytes (17) for disposal in the spleen. Owing to its regulatory role, CR1 (also designated as CD35) appears to limit inflammation and tissue damage (18). Despite these insights, there remains a lack of direct clinical data correlating complement levels with dengue disease severity. Here we quantitatively assessed key complement proteins and a myriad of other clinical laboratory parameters in dengue. We determined if complement factors and acute-phase reactants correlated with disease severity attributing to improved understanding of dengue pathogenesis and endeavored to identify surrogate biomarkers/therapeutic targets in severe dengue.

## Materials and methods

### Ethical approval

**Figure 1:**
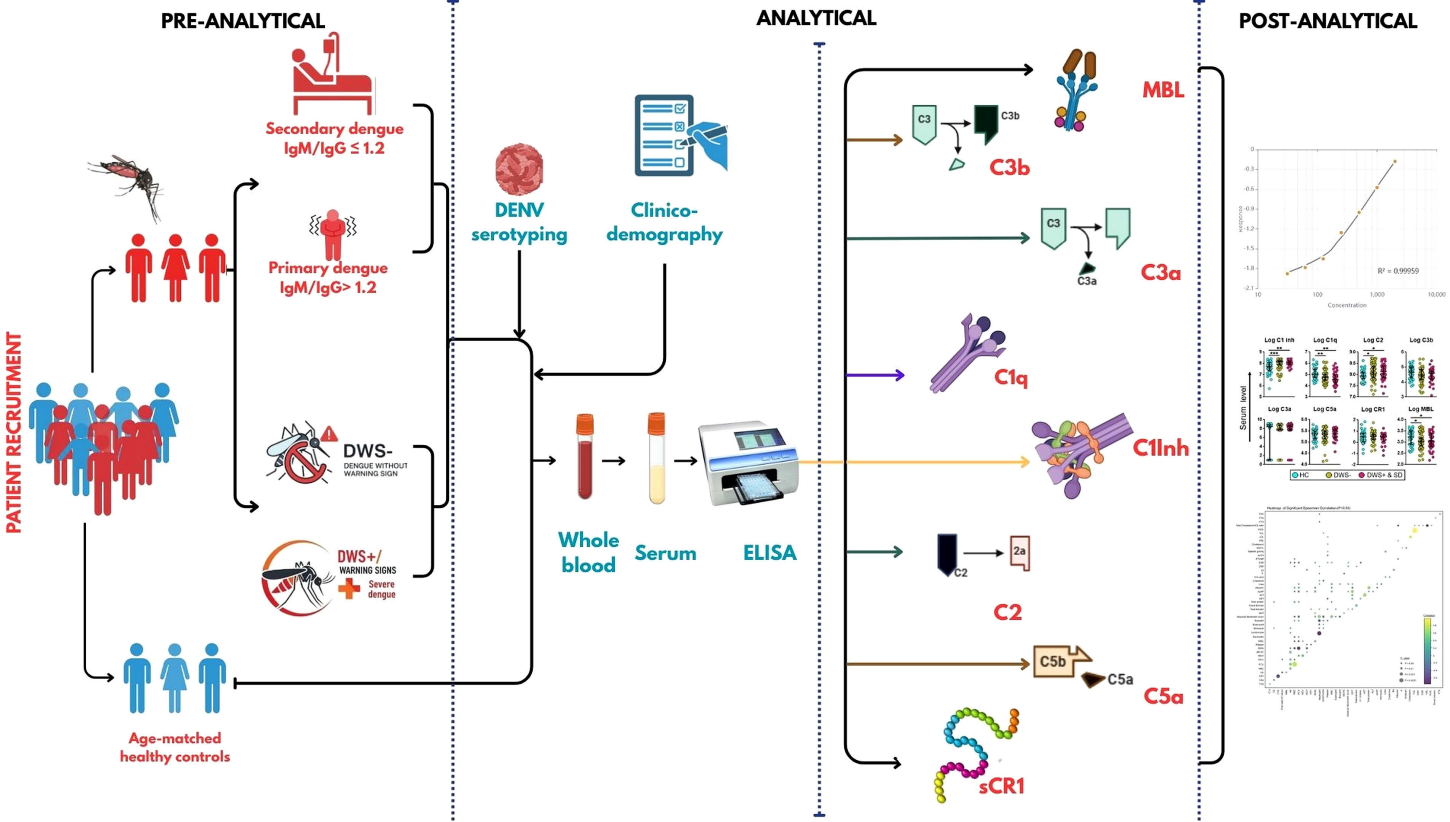
Schematic representation of the work flow in the cross-sectional case–control investigation (visual abstract created using CANVA and Biorender). Complement levels were measured in both primary (1°) and secondary (2°) dengue cohorts as well as across different severities of dengue infection based on the WHO 2007 classification. The serum specimens were collected from patients/volunteers and analyzed using commercial ELISA. C1Inh, C1 inhibitor; DWS-, dengue without warning signs; DWS+, dengue with warning signs; MBL, mannose-binding lectin; sCR1, soluble complement receptor 1.

A prospective, cross-sectional case–control study was carried out in accordance with the Guidelines of the International Conference on Harmonization and the Declaration of Helsinki (1964) to fulfil the study objectives. The Institutional Ethical Committee (IEC) of the Saveetha Medical College and Hospital, Chennai (ref. no. 114/03/2024/Faculty/SRB/SMCH) and the Government Theni Medical College and Hospital, Theni (ref. no. 2300/IEC/2024-26) reviewed all of the protocols and extended necessary approval for conducting the research. Written informed consents were duly obtained from all of the study volunteers/patients. Peripheral blood specimens were collected from the recruited participants in BD Vacutainer (Becton Dickinson, Franklin Lakes, NJ, USA) tubes for the extraction of serum by a trained phlebotomist. The serum aliquots were stored at -80 °C before transporting on dry ice to the study site, where it was maintained at -20 °C until use in the experiments. The schematic representation of the work flow pertinent to the investigations is illustrated in **Figure 1**.

### Dengue diagnosis and clinical classification

This study prospectively recruited febrile patients with a suspicion of clinical dengue at the fever clinic and subsequently admitted at the medical center after confirmation using laboratory methods such as commercial ELISA-based detection of DENV-specific IgM antibodies or NS1 ELISA (19) as dengue infection. NS1 antigen testing was performed at the time of blood collection by the clinicians using a commercial NS1 ELISA kit (01PE40, Abbott, Chicago, IL, USA). Participants recruited at the clinical centers were classified as primary and secondary dengue infections. PanBio Dengue IgM (cat. no.: 01PE20, Abbott, Chicago, IL, USA) and PanBio dengue IgG (cat. no.: 01PE10, Abbott, Chicago, IL, USA) were employed for the characterization of IgM- and IgG-positive clinical samples. The samples were diluted 1:100 in both assays as per the manufacturer’s instructions.

For the anti-DENV IgM assay, samples with a value >11 PanBio units were considered positive, whereas samples with a value >22 PanBio units were considered positive for the anti-DENV IgG assay. Unless and otherwise mentioned, primary dengue infection was defined as those with an IgM/IgG ratio >1.2, and secondary dengue was defined as those with an IgM/IgG ratio ≤1.2 (20). Furthermore, dengue severity was classified using the WHO 2009 classification by specialist clinical practitioners (19). Healthy controls (HCs) were age- and sex-matched volunteers with no history of fever, chronic disease, or recent vaccination in the preceding 4 weeks. All HCs tested negative for dengue IgM or NS1 antigen at the time of enrolment. Blood samples were drawn during the acute phase between 3 and 5 days of dengue onset based on patient history and clinical assessment.

### Quantification of dengue viremia and serotyping

The serum samples were subjected to quantification of DENV viremia using commercial platforms. Briefly, RNA was extracted from serum using a KingFisher™ Flex Purification system (ThermoFisher Scientific, Singapore) using HIPura Prefilled Medium Plates T (HiMedia, Mumbai, India). Subsequently, DENV viral load was quantified on QuantStudio 5 Real Time PCR (Applied Biosystems, Foster City, CA, USA) using a commercial Dengue Real-Time PCR kit (cat. no.: 8013, Helini, Chennai, India) (21). The samples with confirmed viremia were used to identify the DENV serotype using a commercial DENV genotyping real-time PCR kit (cat. no.: 8014, Helini, Chennai, India).

### Chemiluminescence immunoassay

The presence of anti-SARS-CoV-2 IgG was measured in representative samples using a chemiluminescence immunoassay (CLIA) (VITROS 3600, Bridgend, UK). A total of 28 samples were selected randomly for the anti-SARS-CoV-2 IgG antibody measurements. As per the instructions of the manufacturer, the serum samples were pre-diluted with saline solution before measurement using IgG Quantitative reagent pack (cat. no.: 6199960, Vitros Immunodiagnostics, Bridgend, UK).

### Complement ELISA

The products of complement cascade activation were measured by using commercial ELISA. The following serum complement analytes were measured in the study participants as per the instructions of the manufacturers: mannose-binding lectin (MBL) (cat. no.: DMBL00, Biotechne, Minneapolis, USA; sensitivity: 0.002–0.029 ng/mL), C3b (cat. no.: ab195431, Abcam; sensitivity: 73 pg/mL), C3a (cat. no.: ab279352, Abcam; sensitivity: 63 pg/mL), C2 (cat. no.: ab254501, Abcam; sensitivity: 175.11 pg/mL), and C5a (cat. no.: ab193695, Abcam; sensitivity: 31 pg/mL), C1q (cat. no.: ab170246, Abcam; sensitivity: 64 pg/mL), C1 inhibitor (cat. no.: ab224883, Abcam; sensitivity: 10.9 pg/mL), and sCR1/sCD35 (cat. no.: ab277439, Abcam; sensitivity: 0.2 ng/mL).

### Statistical analysis

The comparison of demographic and clinical data was expressed as an interquartile range. The *P* values were calculated using the Chi-square test for categorical variables, while the continuous variables were analyzed using the Kruskal-Wallis test. *P* value <0.05 was considered significant, and was used for the *Post hoc* Mann-Whitney U test. Univariate regression models were used to explore significant relationships with outcome variables, which were subsequently included in the multivariate model. The Hosmer–Lemeshow values for the binary model and linear model were *P* = 0.443 and 0.851, respectively. *P*-values <0.05, <0.01, <0.001, <0.0001 were marked as ∗, ∗∗, ∗∗∗, and ∗∗∗∗, respectively. All of the analyses were performed using PRISM Software Ver.6.0 (GraphPad, CA, USA) and SPSS 20.0 (IBM Corp, New York, USA).

## Results

### Clinico-demographic characteristics of the study cohort

The present study recruited 156 participants that encompassed DENV-infected patients (*n* = 114) and HCs (*n* = 42). Serological investigations (by commercial ELISA) revealed 92% participants (*n* = 104) positive for anti-DENV IgM, 31% (*n* = 35) positive for anti-DENV IgG, and 21.2% (*n* = 24) positive for DENV NS1, and 18 samples showed positivity for both DENV NS1 antigenemia and anti-DENV IgM levels. Based on the criteria discussed in the methodology, 26 patients were identified as secondary dengue infection cases for the study (**Table 1**). Of the 114 dengue-positive cases, 47.4% (*n* = 74) patients were classified under DWS-, 22.4% (*n* = 35) were categorized under DWS+, and 3.2% (*n* = 5) were included under severe dengue.

**Table 1:** Patients’ characteristics for demography, laboratory, and serum complement analytes at admission.

Demographic and laboratory parameters	Total	Healthy controls	Clinical dengue	*P*-value
Age, years	30 (23, 40)	27 (22, 32.5)	32 (25, 42)	0.004**
Sex, male, *n* (%)	111 (71.2)	42 (100)	69 (61.1)	<0.0001
Dengue category				
DWS-	--	--	74 (47.4%)	--
DWS+	--	--	35 (22.4%)	--
Severe dengue	--	--	5 (3.2%)	--
IgM positive, *n* (%)	--	--	104 (92%)	--
IgM titer	--	--	27.9 (15.4, 57.8)	--
IgG positive, *n* (%)	--	--	35 (31%)	--
IgG titer	--	--	10.5 (4.9, 50)	--
IgM/IgG ratio	--	--	2 (0.9, 4.52)	--
2° infection, *n* (%)	--	--	26 (23%)	--
NS1 positive, *n* (%)	--	--	24 (21.2%)	--
C1Inh, pg/mL	8.3 (7.78, 8.39)	7.95 (7.5, 8.3)	8.3 (8, 8.4)	0.001**
C1q, ng/mL	4.77 (4.4, 5.08)	4.9 (4.75, 5.48)	4.64 (4.3, 5.01)	0.001**
C2, pg/mL	8.08 (7.9, 8.35)	8 (7.87, 8.16)	8.11 (7.93, 8.43)	0.014*
C3a, pg/mL	8.4 (7.69, 8.73)	8.45 (1, 8.78)	8.35 (7.72, 8.73)	0.013*
C3b, ng/mL	4.52 (4.2, 4.81)	4.7 (4.32, 5)	4.45 (4.15, 4.75)	0.718
C5a, pg/mL	5.36 (5.16, 5.49)	5.37 (5.16, 6.49)	5.36 (5.17, 5.5)	0.992
sCR1, ng/mL	0.47 (0.03, 0.72)	0.48 (0.05, 0.8)	0.47 (0.02, 0.69)	0.509
MBL, ng/mL	3.04 (2.84, 3.36)	3.21 (2.9, 3.49)	3.02 (1.21, 4.58)	0.023*

Comparison of demographic and clinical characteristics between dengue patients and healthy controls (HCs). All data are expressed as median (IQR) unless specified. *P*-values are calculated by using chi-square test for categorical variables and Kruskal–Wallis test for continuous variables.

IQR, interquartile range; DWS-, dengue without warning signs; DWS+, dengue with warning signs; MBL, mannose-binding lectin.

P-value <0.05 (significant); *, <0.05; **, <0.01; ***, <0.001.

Viral load estimation was done for 80 samples, of which only nine samples were positive for dengue viremia. All nine DENV viremia samples were concurrently positive for DENV NS1, three were positive for both NS1 and IgM, and one was identified as secondary dengue. DENV serotyping was successful only in three of the viremic samples, and all were identified as DENV2. Similarly, of the 28 representative serum samples measured for SARS-CoV-2 IgG levels, all were observed to be reactive (cutoff for high positive >139 BAU/mL), with levels as high as 200 BAU/mL in all of the samples examined. SARS-CoV-2 IgG positivity was not significantly associated with dengue severity (95% CI = 1.435 (0.566–3.642); *P* = 0.44).

### Complement factors C1q, C1Inh (C1 esterase), and C2 were differentially expressed and varied between grades of dengue severity

**Figure 2:**
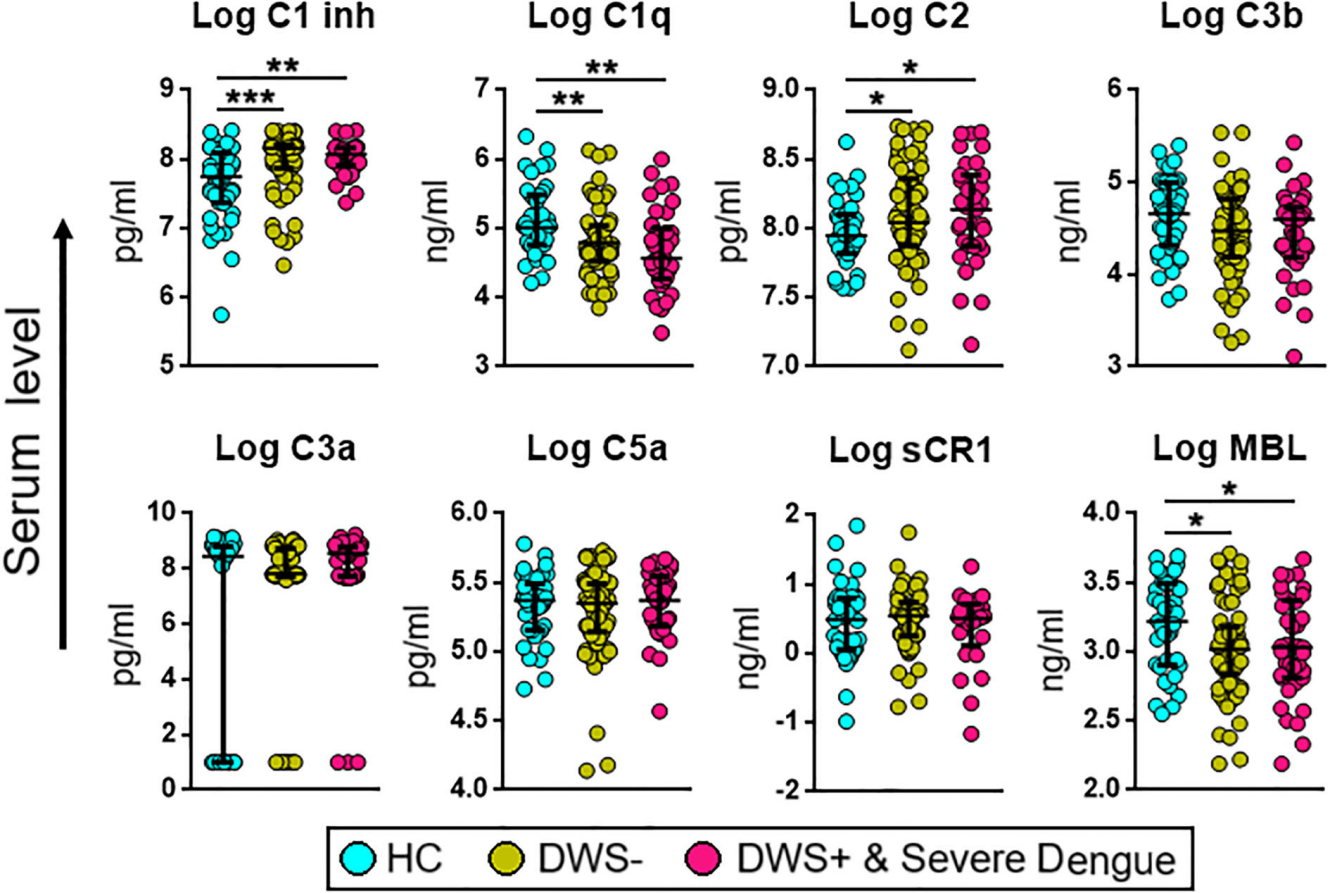
Comparison of serum levels of complement proteins in healthy controls (HCs), dengue without warning signs (DWS-) as well as dengue with warning signs (DWS) and severe dengue. Levels of biomarkers were compared across the three study groups by Kruskal–Wallis test. *Post hoc* Mann–Whitney *U*-tests were subsequently performed for those complement biomarkers with a Kruskal–Wallis *P*-value of <0.05. *P*-value <0.05 (significant); *, <0.05; **, <0.01; ***, <0.001. HC, healthy controls; DWS-, dengue without warning signs; DWS+, dengue with warning signs; C1Inh, C1 inhibitor; sCR1, soluble complement receptor 1; MBL, mannose-binding lectin.

The analysis of complement components across the stages of DENV infection revealed that C1Inh levels were statistically significant between dengue patients and HCs (*P* < 0.01) and between HCs and primary dengue (*P* < 0.001) cohorts. The levels of C1q between dengue patients and HCs (*P* < 0.01) and between HCs and primary dengue (*P* < 0.01) were also statistically significant. We also found marked differential levels in C2 between dengue patients and HCs (*P* < 0.5) and between HCs and primary dengue (*P* < 0.05) cohorts. MBL showed a mild but significant variation (*P* < 0.05), suggesting a partial engagement of the lectin pathway (**Figure 2**). Conversely, activation products C3b, C3a, and C5a and the regulatory complement protein sCR1 did not vary significantly across groups in this dataset, which indicates that downstream complement activation may not be uniformly altered during disease advancement (*P* > 0.5).

### Expressions of C1Inh, C1q, and C2 were significantly altered across primary and secondary dengue patients

**Figure 3:**
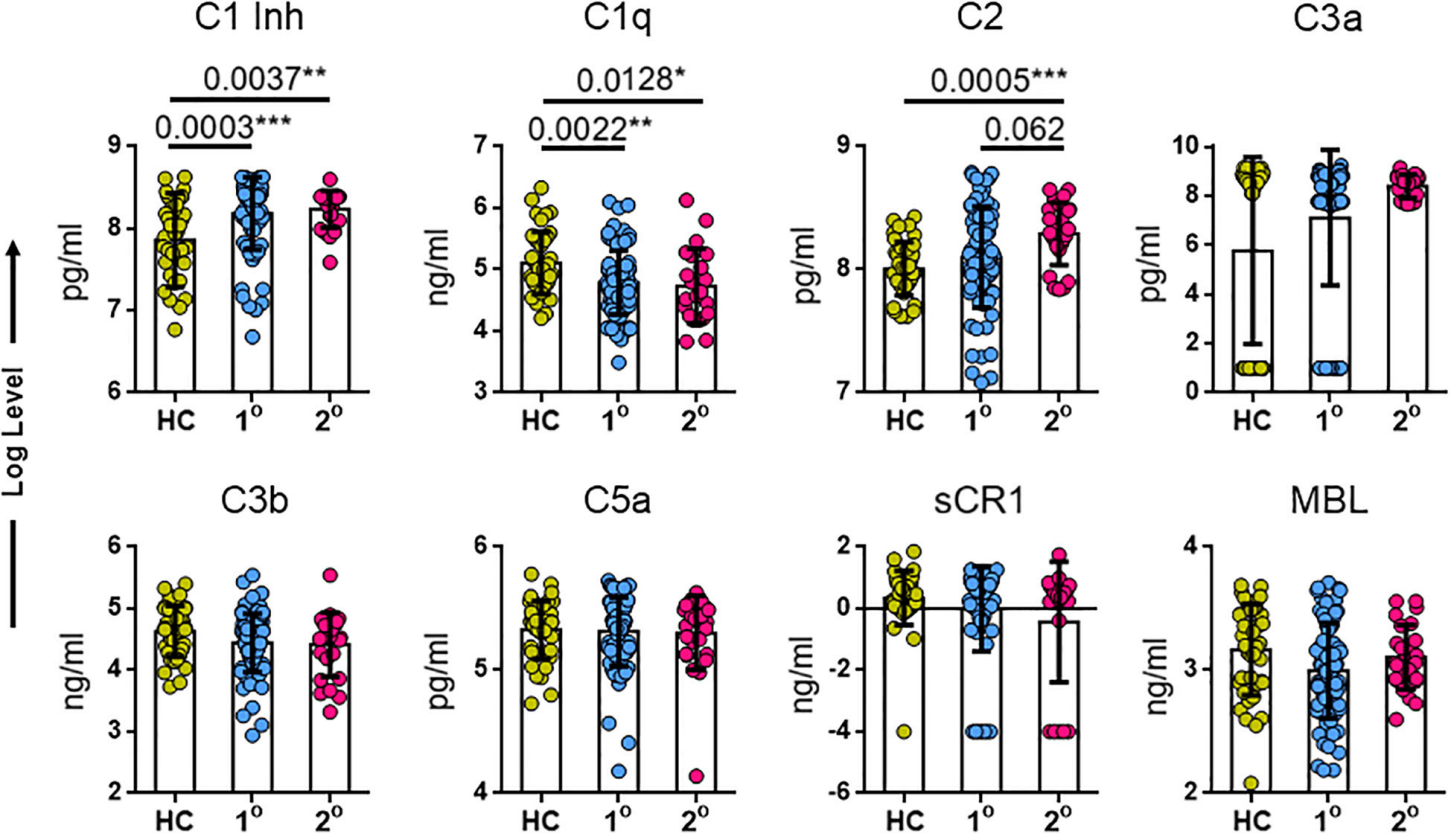
Comparison of serum levels of complement markers in healthy controls (HCs), 1° refers to patients with primary dengue and 2° to individuals with secondary dengue infections. Levels of biomarkers were compared across the three patient groups by Kruskal–Wallis test. *Post hoc* Mann–Whitney *U*-tests were subsequently performed for those biomarkers with a Kruskal–Wallis test *P*-value of <0.05. *P*-value <0.05 (significant); *, <0.05; **, <0.01; ***, <0.001. HCs, healthy controls; C1Inh, C1 inhibitor; sCR1, soluble complement receptor 1; MBL, mannose-binding lectin.

Having said that the levels of C1q, C1Inh, and C2 are varied across grades of dengue severity, we studied next if their expressions varied between primary and secondary dengue patients. A comparative analysis of complement among primary and secondary dengue patients and HCs revealed a selective pattern. Early complement proteins including C1Inh levels showed statistical significance between dengue patients and HCs (*P* = 0.0037) and between HCs and primary dengue (*P* = 0.0003) cohorts. The C1q levels between dengue patients and HCs (*P* = 0.0128) and between HCs and primary dengue (*P* = 0.0022) also revealed statistical significance (**Figure 3**). The C2 levels between dengue patients and HCs (*P* = 0.0005) and between HCs and primary dengue (*P* = 0.062) were markedly altered across patient groups, while activation products C3b, C3a, and C5a and regulatory protein, sCR1 (*P* > 0.5), did not vary significantly across groups in this dataset, which is consistent with its expression in the severity. MBL did not show any significant variation between the groups unlike in disease severity (**Figure 3**).

### Every unit of increase in soluble CR1 levels was associated with 22% reduced odds of dengue disease severity

**Figure 4:**
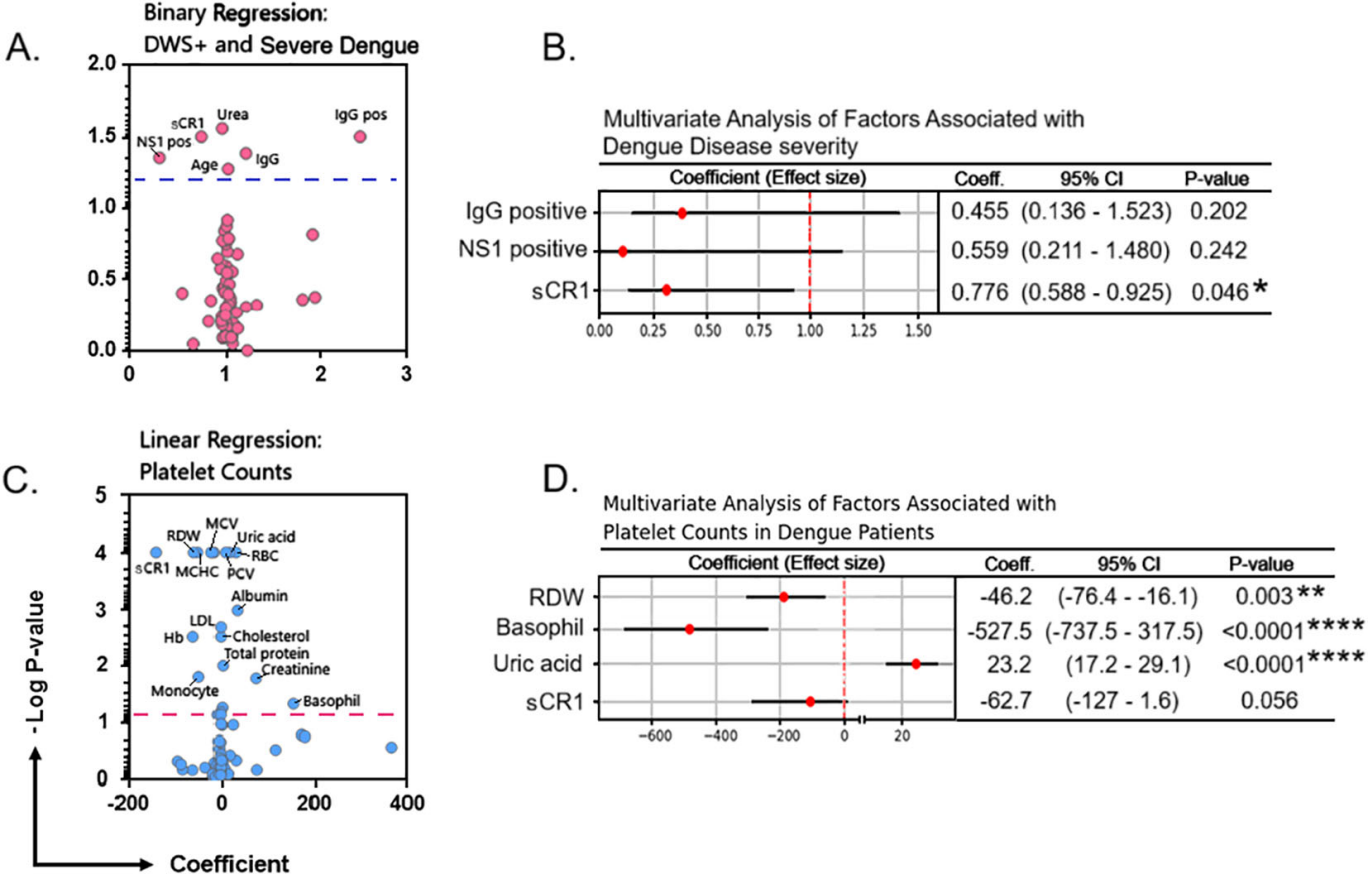
Factors that are associated with dengue disease severity and platelet counts. **(A)** Binary regression analysis of factors associated with the severity of dengue disease (DWS+ and severe dengue). **(B)** Multivariate analysis of factors associated with dengue disease severity. **(C)** Linear regression analysis of factors associated with blood platelet counts in dengue patients. **(D)** Multivariate analysis of factors associated with platelet counts in dengue patients. By using a univariate regression model, variables that showed a significant relationship with outcome variables (i.e., development of dengue with warning signs and or blood platelet counts) in the univariate model will then be included in the multivariate model. The Hosmer–Lemeshow values for binary model and linear model were *P* = 0.443 and *P* = 0.851, respectively. *P*-value <0.05 (significant); *, <0.05; **, <0.01; ****, <0.0001. Hb, hemoglobin; LDL, low-density lipoprotein; MCHC, mean corpuscular hemoglobin concentration; MCV, mean corpuscular volume; PCV, packed cell volume; RBC, red blood cell; RDW, red cell distribution width; sCR1, soluble complement receptor 1.

Next, we used univariate regression models to unveil significant relationships with outcome variables, which were subsequently included in a multivariate model. Univariate analysis revealed that sCR1, NS1 positivity, age, urea, and IgG positivity were significantly associated with dengue severity. However, in the multivariate logistic analysis, only sCR1 showed a significant association with disease severity, where this was associated with reduced odds of dengue severity by 22% (coef. = 0.776, 95% CI = 0.588–0.925; *P* = 0.046), suggesting a potential protective role (**Figures 4A, B**).

### Serum uric acid levels were directly correlated with blood platelet levels in clinical dengue infection

We assessed a wide range of hematologic and biochemical parameters/analytes for their relationship with blood platelet counts. Significant negative associations were observed with red cell distribution width (RDW) and peripheral basophil counts (*P* = 0.003 and *P* < 0.0001, respectively), whereas sCR1 showed a negative trend but was not statistically significant (*P* = 0.056). Concurrently, the levels of serum uric acid showed a strongly positive correlation with blood platelet counts (*P* < 0.0001) (**Figures 4C, D**) by a multivariate analysis.

### Complement anaphylatoxins C3a and C5a showed significant correlations with immune markers and dengue viremia

**Figure 5:**
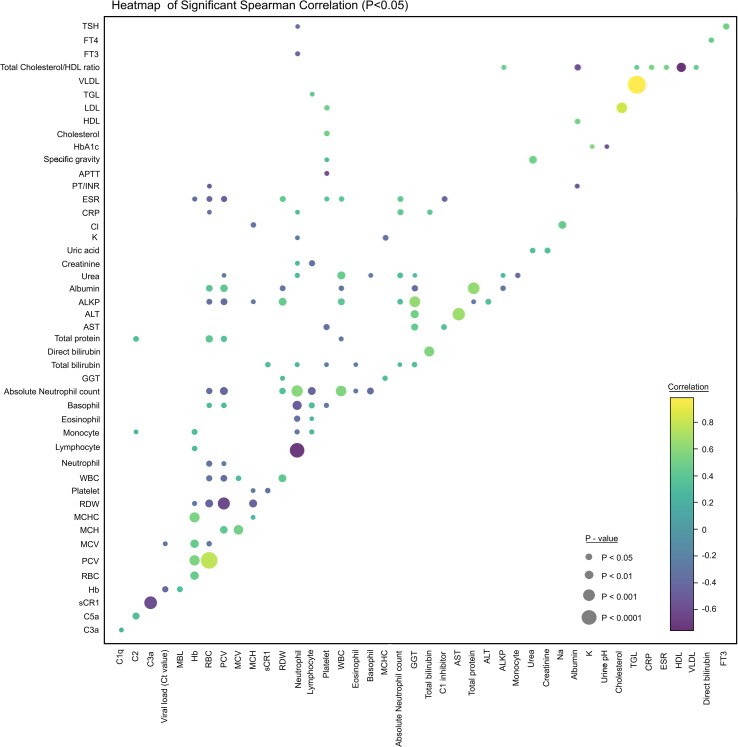
Heatmap of significant Spearman correlations (*P* < 0.05) among clinical, biochemical, hematologic, and immunological parameters. Warm color indicates positive correlation, dark color indicates negative correlation, and bubble size represents the level of significance. ALKP, alkaline phosphatase; ALT, alanine aminotransferase; APTT, activated partial thromboplastin time; AST, aspartate aminotransferase; CRP, carbohydrate reactive protein; ESR, erythrocyte sedimentation rate; FT3, free triiodothyronine, FT4, free thyroxine; GGT, gamma glutamyl transpeptidase; Hb, hemoglobin; HbA1c, glycosylated hemoglobin; HDL, high-density lipoprotein; LDL, low-density lipoprotein; MCHC, mean corpuscular hemoglobin concentration; MCV, mean corpuscular volume; PCV, packed cell volume; PT/INR, prothrombin time/international normalized ratio; RBC, red blood cell; RDW, red cell distribution width; sCR1, soluble complement receptor 1; TGL, triglyceride; TSH, thyroid-stimulating hormone; VLDL, very-low-density lipoprotein; WBC, white blood cell.

Spearman correlation analysis revealed strong positive correlations with liver function markers ALT, AST, ALKP, GGT, and bilirubin with each other. On the other hand, lipid profile components, for instance, low-density lipoproteins (LDL), high-density lipoproteins (HDL), triglycerides (TGL), very-low-density lipoproteins (VLDL), and cholesterol ratios showed both positive and negative correlations depending on the parameters. We also found correlations of certain acute phase inflammatory markers such as C-reactive protein (CRP) and erythrocyte sedimentation rate (ESR) with neutrophils and lymphocytes. Certain renal function markers like creatinine, urea, and uric acid showed inter-correlations and some associations with plasma potassium and chloride electrolyte levels. We also found that the complement anaphylatoxins C3a, C5a, and C1q show correlations with immune markers and possibly DENV viral load (**Figure 5**).

## Discussion

In the current cross-sectional case–control investigation, we surveyed the role of various clinical laboratory parameters and immunological determinants, with particular emphasis on the complement system in DENV across varying grades of disease severity. Of the 156 study participants, 114 cases were serologically confirmed cases of DENV infection, with a predominance of IgM positivity and a small number of NS1-positive and secondary dengue cases. Viral RNA was also present in a minority of samples, all of which were NS1-positive, reinforcing the utility of NS1 as a marker of acute dengue viremia (22, 23). The low percentage of samples with dengue viremia could be attributed to the temporospatial inaccuracies in specimen collection (likely during the late acute phase (between days 3 and 5 of the onset of fever) when viremia has typically reduced, rather than assay failure. This is consistent with established dengue kinetics, where viral RNA falls rapidly in 4 to 5 days of illness (24).

Analysis of complement components across stages of infection revealed distinct alterations in the early classical complement proteins. C1Inh (regulatory), C1q, and C2 demonstrated severity-dependent variations, suggesting that the classical pathway plays a crucial role in dengue immunopathogenesis. Our observations are consistent with prior evidence indicating that the formation of an immune complex and antibody-mediated response can trigger the activation of a classical pathway in dengue (25, 26). Antibody-dependent enhancement (ADE) of dengue virus infection has been one of the main hypotheses to explain the disease severity of dengue (26). In the presence of sub-neutralizing antibody, immune complexes can amplify the viral replication in phagocytic cells, which will, in turn, activate the complement system via cytokine cascade (9, 27). The elevated levels of C1Inh likely represent a host’s compensatory response aimed at counteracting excessive complement activation, thereby limiting the inflammation and the likely vascular activation mechanisms triggered (28). Similarly, the attrition in C1q levels is consistent with its consumption during immune-complex-driven activation, a well-defined hallmark of dengue disease, especially during secondary infection (26, 29).

Our study also observed that MBL did not have any significant differential expression across primary and secondary dengue groups, although it did show a differential expression between varying grades of dengue disease severity. This observation, in turn, is consistent with the functional biology of MBL as a lectin complement pathway protein that recognizes carbohydrate moieties (23, 30). Thus, the expression of MBL is not expected to vary remarkably between primary and secondary dengue, which primarily differentiates between humoral immune responses. On the contrary, the association of MBL with disease severity highlights its role in modulating host–virus interactions and complement activation during acute infection, making it more marker-associated with clinical outcome and not of disease severity (17, 30). Our investigations also showed that the downstream complement activation products, i.e., C3a, C3b, and C5a, and the complement regulatory protein sCR1 did not show any significant difference in expression in varying grades of disease severity, i.e., DWS+, DWS-, and severe dengue as well as primary/secondary dengue. This suggests that terminal complement activation is not uniformly altered during disease severity, possibly due to efficient regulation or localized activation.

The selective alterations in C1q, C1Inh, and C2 levels in overall patient cohort point to a preferential engagement of the classical pathway during early DENV infection. Significant shifts in the proximal classical complement factors and the lack of marked alterations in downstream components suggest the likely initiation of the classical pathway but not fully propagated across to the terminal effectors of complement activation (involving C6, C7, C8, and C9, which, however, could not be completely explored in the current study). This could still be speculated of the likely involvement of early immune-complex-driven activation but are tightly regulated, which remains to be explored. In contrast, a minimal variation in MBL in primary/secondary dengue cohort while indicating significant alterations in the dengue cohort indicates that the lectin pathway may seldom contribute to complement activation. This supports a suggestive interplay, where classical pathway may likely serve as the main axle of complement activation in DENV infection (17, 31).

One of the most important observations was the association between sCR1 and disease severity. By univariate analysis, several parameters, including age, NS1 positivity, and IgG positivity, were found to be associated with dengue severity, while multivariate modeling identified sCR1 as the only factor associated with dengue disease severity. Furthermore, higher sCR1 levels were protective, with each unit increase of sCR1 reducing the dengue severity by 22%. This suggests that sCR1 exhibits a regulatory role in controlling complement activation and preventing excessive inflammation and tissue injury during systemic infection (26, 32). Many previous studies have implicated uncontrollable complement activation as a contributor to plasma leakage, and our study hypothesizes the role of sCR1 as a protective regulator (33). In addition, sCR1 showed a negative association with platelet count but which did not reach statistical significance, implicating that its suggestive role is more closely related to dengue severity than to thrombocytopenia. It is important to note that the complement system also links to the coagulation systems, and hence the association between sCR1 and platelet requires greater attention. CR1 is a type I membrane protein that encompasses a family of complement receptors (CR2, CR3, and CR4) that are often expressed on a plethora of immune cells (34). Besides that, very low levels (~50 ng/mL) of CR1 occur as sCR1 in the plasma, likely released as a result of proteolytic shedding (34).

Hematologic and biochemical results associated with the platelet count also provided additional insights. In line with previous findings (35, 36), markers of immune dysregulation, such as RDW and basophil levels, were inversely correlated with the platelet levels. These findings are consistent with the ongoing hematologic derangement occurring in dengue thrombocytopenia (35, 36). Interestingly, uric acid levels showed a strong positive correlation with platelet count, raising the probability that altered purine metabolism or oxidative stress pathway can influence the hematologic recovery in DENV infection (37). However, this observation is only preliminary and requires further in-depth investigations. Uric acid functions as a major endogenous antioxidant, scavenging ROS and protecting the host against cellular damage (38). The observed positive correlation between uric acid and platelet levels and its implied negative correlation between dengue severity reflect excessive oxidative stress and antioxidant depletion, implying an uptake of uric acid metabolites and a decreased capacity of endogenous antioxidant defense due to disease severity (38).

The study has certain limitations that should be acknowledged. Potential confounders such as prior flavivirus infection, nutritional status, and other co-morbidities were not fully controlled. Although all of the representative samples tested were positive for SARS-CoV-2 IgG, reducing intergroup variability related to COVID-19 exposure; this had no association with dengue severity. The relatively small sample size might limit the statistical power and generalizability of the findings. Although plaque reduction neutralization test (PNRT) helps in confirming DENV infection, this was not performed due to sample volume constraints. In addition, although we accessed complement activation, not all complement components (e.g., C4, C5b, C6, C7, C8, and C9) were measured. Furthermore, the cross-sectional study design limits the establishment of the causal relationship between the target analyte and disease exacerbation or mitigation over the period of disease progression, warranting longitudinal follow-up investigations to capture temporospatial disease dynamics. Moreover, high-throughput omics-based approaches such as transcriptomics, proteomics, metabolomics, and other validation experiments to identify relevant disease mediators or therapeutic targets are warranted. Lastly, the limited viremia positivity among the samples and restricted serotyping constrain the generalizability of serotype-dependent complement alterations. Taken together, these limitations suggest that the current findings should rather be considered as indicative, and further large-scale, longitudinal investigations encompassing all of the complement factors are warranted to validate the findings.

In conclusion, our investigation reinforces the importance of the complement system in dengue pathogenesis, with particular emphasis on the early classical pathway of complement activation. Furthermore, the association of sCR1 indicates its likelihood for use as a potential candidate biomarker of dengue severity, which, however, remains to be confirmed using longitudinal cohort studies. Given the strong positive correlation of serum uric acid with blood platelet counts, the relevance of uric acid (an endogenous antioxidant) can be elaborately studied in dengue. This study offers several avenues for future translational research aimed at improving dengue diagnostic, highlighting the complement factors’ potential utility as biomarker candidates in distinguishing disease severity or predicting clinical progression. Future studies incorporating longitudinal sampling, comprehensive profiling of terminal pathways, and larger, virologically diverse cohorts will be essential to validate the pathways explored herein for rendering translational validations in clinical dengue infection.

## Data Availability

The raw data supporting the conclusions of this article will be made available by the authors, without undue reservation.
